# Survival benefit from liver transplantation for patients with and without hepatocellular carcinoma

**DOI:** 10.1016/j.jhepr.2023.100907

**Published:** 2023-09-13

**Authors:** Ben F.J. Goudsmit, Ilaria Prosepe, Maarten E. Tushuizen, Vincenzo Mazzaferro, Ian P.J. Alwayn, Bart van Hoek, Andries E. Braat, Hein Putter

**Affiliations:** 1 Department of Gastroenterology and Hepatology, Leiden University Medical Center, Leiden, The Netherlands; 2 Department of Biomedical Data Sciences, Leiden University Medical Center, Leiden, The Netherlands; 3 Transplant Center, Leiden University Medical Center, Leiden, The Netherlands; 4 Department of Oncology, University of Milan, Milan, Italy; 5 Hepatology and Liver Transplantation Unit, Department of Surgery, Fondazione Istituto di Ricovero e Cura a Carattere Scientifico, Istituto Nazionale Tumori, Milan, Italy; 6 Department of Surgery, Leiden University Medical Center, Leiden, The Netherlands

**Keywords:** Liver transplantation, Survival benefit, Hepatocellular carcinoma

## Abstract

**Background & Aims:**

In the USA, inequal liver transplantation (LT) access exists between patients with and without hepatocellular carcinoma (HCC). Survival benefit considers survival without and with LT and could equalise LT access. We calculated bias-corrected LT survival benefit for patients with(out) HCC who underwent a transplant, based on longitudinal data in a recent United States cohort.

**Methods:**

Adult LT candidates with(out) HCC between 2010 and 2019 were included. Waitlist survival over time was contrasted to post-transplant survival, to estimate 5-year survival benefit from the moment of LT. Waitlist survival was modelled with a bias-corrected Cox regression, and post-transplant survival was estimated through Cox proportional hazards regression.

**Results:**

Mean HCC survival without LT was always lower than non-HCC waitlist survival. Below model for end-stage liver disease (sodium) (MELD(-Na)) 30, patients with HCC gained more life-years from LT than patients without HCC at the same MELD(-Na) score. Only patients without HCC below MELD(-Na) 9 had negative benefit. Most patients with HCC underwent a transplant below MELD(-Na) 14, and most patients without HCC underwent a transplant above MELD(-Na) 26. Liver function [MELD(-Na), albumin] was the main predictor of 5-year benefit. Therefore, during 5 years, most patients with HCC gained 0.12 to 1.96 years from LT, whereas most patients without HCC gained 2.48 to 3.45 years.

**Conclusions:**

On an individual level, performing a transplant in patients with HCC resulted in survival benefit. However, on a population level, benefit was indirectly decreased, as patients without HCC were likely to gain more survival owing to decreased liver function. For patients who underwent a transplant, a constructed online calculator estimates 5-year survival benefit given specific patient characteristics. Survival benefit scores could serve to equalise LT access.

**Impact and implications:**

Benefit is a comparison of the survival with and without liver transplantation, and it is important when deciding who should undergo a transplant. Liver function is most important when predicting possible benefit from transplantation. Patients with liver cancer die sooner on the waiting list than similar patients without liver cancer. However, patients with liver cancer more often have better liver function. Most patients without liver cancer derive more benefit from transplantation than patients with liver cancer.

## Highlights

• Over 50% of patients without HCC underwent a transplant above MELD(-Na) 26.
• Over 50% of patients with HCC underwent a transplant below MELD(-Na) score 14.
• The average 5-year benefit of patients without HCC was 2.3 years.
• For patients with HCC, this was 1.19 (within the Milan criteria) and 1.45 (initially outside the Milan criteria) years.
• On a population level (over)prioritising patients with HCC can indirectly waste transplant benefit.

## Graphical abstract

**Figure:**
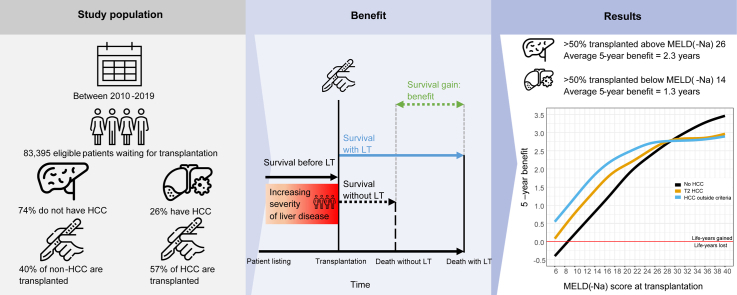

## Introduction

Liver transplantation (LT) relies on scarce donor grafts. Therefore, USA liver allocation prioritises patients who likely will die soonest without transplantation,^1^ expressed through model for end-stage liver disease sodium (MELD-Na) scores.^2^^,^^3^ Because MELD-Na fails to adequately predict survival in some patients, most notably those with hepatocellular carcinoma (HCC),^4^ exception points have been used for LT allocation instead,[5], [6], [7] which unintendedly increased HCC LT access too much.[8], [9], [10] Therefore, inequality on the LT waiting list exists and the need for LT is expressed differently for patients with HCC and those without HCC.

**Fig. 1:**
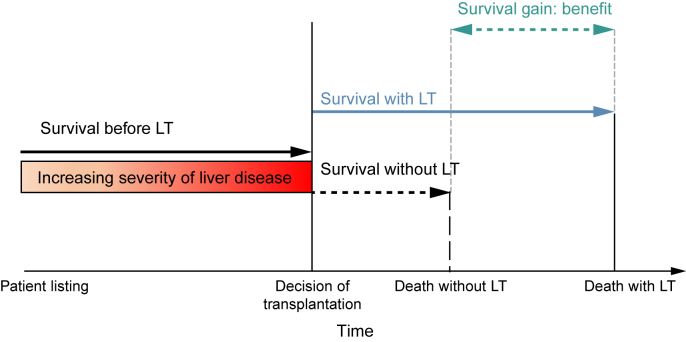
Illustration of benefit: life-years gained through transplantation. Survival benefit is defined as the difference in 5-year life expectancy with and without transplantation. Although patients are waiting for LT, time passes and disease severity typically changes. At the moment of transplantation, benefit is estimated. The survival up until transplantation (‘survival before LT’) is used to predict waiting list survival in the absence of transplantation (‘survival without LT’). Without LT survival is then contrasted to posttransplant survival (‘with LT’) to calculate benefit. Note that the dashed lines represent outcomes that are not observed in patients who underwent a transplant. LT, liver transplantation.

As an equalising principle, LT survival benefit could be used, which is the difference between survival with and without transplantation.^11^ Considering LT survival benefit is valuable because donor grafts are scarce and some patients gain more life-years than others.[11], [12], [13], [14] Considering benefit also better approximates clinical decision-making at liver graft offering. Perhaps for these reasons, LT benefit has been studied before.^9^^,^[15], [16], [17], [18] Patients possibly gain survival from the moment of LT (Fig. 1). Therefore, to calculate benefit, future survival with and without LT must be estimated from the moment of LT. However, previous studies used (1) only first listing data to (2) calculate waiting list survival up until the moment of LT (Fig. 1, ‘before LT survival’).^9^^,^[15], [16], [17], [18] This possibly is a suboptimal approximation of survival on the waiting list, as liver graft acceptance on average lies 6 to 8 months beyond the moment of first listing.^19^ During this time, liver disease typically progresses,^20^^,^^21^ patients can drop out,^19^ or HCC could be downstaged.^22^ This changes survival rates as compared with first listing,^11^^,^^12^^,^^14^^,^^23^ which should be accounted for.

As an alternative, we propose to (1) use longitudinal waiting list data to (2) estimate benefit from the moment of possible transplantation, hypothesising that this improves representation of the actual waiting list. Furthermore, patient characteristics beyond MELD-Na and exception points will be used to model the risk of waiting list dropout.^6^^,^^10^^,^^15^^,^^17^^,^^24^ Therefore, in a large and recent United States cohort, this study aims to construct a single benefit score for patients with and without HCC. We define benefit as the difference between post-transplant and waiting list survival from the moment of transplantation. Because of the inequal LT access, survival benefit will also be compared between patients with and without HCC. Lastly, benefit scores will be made readily available through an online calculator, to aid clinical decision-making.

## Patients and methods

The TRIPOD (Transparent Reporting of a multivariable prediction model for Individual Prognosis or Diagnosis) statement was used for reporting this study.^25^

### Patient population

This retrospective cohort analysis included adult (≥18 years of age) patients listed for a first LT between 1 January 2010 and 30 April 2019 on the United Network for Organ Sharing (UNOS) waiting list (Fig. S1). This interval ended before the 14 May 2019 implementation of median MELD at transplant.^26^ It also compromised the most recent data with adequate 5-year follow-up completeness. We aimed to calculate benefit for two patient groups: patients without HCC and without exception points (non-HCC group), and patients with HCC and with exception points (HCC group). Although other diseases also qualify for exception points, such as primary sclerosing cholangitis and biliary cirrhosis, we only assessed patients with HCC, as this is by far the largest group and incidence is increasing.^19^ Current Organ Procurement and Transplantation Network (OPTN) policy allows standard exception points for (1) patients with HCC within the Milan criteria (henceforth T2 HCC)^27^ and (2) patients with HCC initially outside the Milan criteria but successfully downstaged within the criteria through locoregional treatment before listing (henceforth HCC outside the criteria). Although a previous study found that outcomes of these groups were similar,^28^ we separately analysed these groups, as the initial HCC disease severity and non-LT treatment are different. We excluded patients with previous LT (n = 4,763; 4.7%), acute liver failure (n = 2,459; 2.5%), listing for living donation (n = 2,116; 2.1%), non-HCC malignancy (n = 783; 0.8%), listing for multiple organs (n = 839; 0.8%), and non-HCC exceptions (n = 6,962; 6.9%) (Fig. S1). We randomly split our population in training data (67% of patients) and validation data (the remaining 33% of patients).

### Benefit definition

Survival benefit was defined as the life-years gained from the moment of transplantation during the next 5 years (Fig. 1).^12^^,^^29^ Survival benefit was calculated by contrasting patient survival in the absence of transplantation (‘without LT’ survival) to post-transplant survival (‘with LT’). To estimate the ‘without LT’ survival, sequential trials together with inverse probability of censoring weighting (IPCW) were used.[12], [13], [14]^,^^23^^,^[30], [31], [32] Supplement S1 includes further methodological details and motivation.

### Statistical analysis

#### Waiting list survival

After dividing the waiting list population in biweekly cross-sections,^12^ repeated MELD or MELD-Na scores were modelled via a Cox proportional hazards regression, respectively, before or after 11 January 2016. Additional predictors were used to correct the longitudinal data (Table S1), which were selected from available UNOS candidate variables deemed clinically relevant in published studies.^6^^,^^12^^,^^15^^,^^17^^,^^24^ Some variables were excluded *a priori*, because they referred to paediatric recipients, exclusion criteria, or donor characteristics. For patients with HCC, date and type of pre-LT treatments were specifically included to account for their effects on waiting list survival (see Table S1). The outcome of analysis was waiting list mortality, which comprised death while awaiting LT and removal because of worsened condition. We censored for all other outcomes (*e.g.* transplantation, removal as a result of recovery, and end of study) and corrected for dependent censoring with IPCW.

#### Post-transplantation survival

Cox proportional hazards regression was used to model post-transplant survival. Predictors were selected by assessing relations of available UNOS recipient and donor variables to 5-year survival in univariate models, with backwards selection of significant variables in multivariate analysis. The outcome was 5-year post-transplant survival, defined as the difference between the date of transplantation and the earliest date of death, or censoring as a result of loss to follow-up or end of study on 30 April 2019.

#### Calculating benefit scores

After establishing the Cox models in the training data, 5-year survival benefit from LT was calculated for each patient who underwent a transplant in the independent validation data. Benefit scores were averaged per biochemical MELD or MELD-Na score at transplantation and stratified for patients without and with HCC. Average benefit was visualised with smoothed plots per MELD(-Na) score and (non-)HCC disease. Model discrimination for 5-year survival was assessed by calculating the area under the receiver operating characteristic curve (AUROC). Cox proportional hazards model calibration (*i.e.* model accuracy) at 5 years was assessed based on bootstrapping with 200 repetitions, to obtain overfitting-corrected estimates of predicted survival, which were compared with observed survival probabilities.^33^

#### Online benefit score calculator

It was of interest to calculate LT benefit scores based on individual patient and donor characteristics. Therefore, we fit a regression model to the calculated 5-year survival benefit scores. To compromise clinical ease of use and predictive power, only the most predictive variables were used in the benefit regression model. Variable importance for benefit prediction was assessed through ANOVAs. We used the overfitting-corrected R^2^ to assess how much variation in benefit was explained by the predictors.^33^ An R^2^ value of 1 indicates that all variability in predictions is accounted for. An R^2^ value above 0.9 indicates excellent model predictions. The online calculator gives graphical summaries of benefit, averaged per MELD-Na score and (non-)HCC disease, to illustrate the gain of life-years during the next 5 years. Of note, the calculator should be used only to estimate benefit for patients who underwent a transplant meeting inclusion criteria.

## Results

### Patient characteristics at transplantation

Characteristics for patients without and with HCC at transplantation between 2010 and 2019 are shown in Table 1. Compared with patients without HCC, those with HCC were slightly older, more often male, and less often of White race/ethnicity. Patients with HCC also more frequently had diabetes mellitus, were less dependent on renal replacement therapy, and had lower median MELD(-Na) scores. Patients with HCC mostly underwent a transplant in medium (2, 4, 6, 7, and 8) and long (1, 5, and 9) UNOS waiting time regions, whereas patients without HCC mostly underwent a transplant in short (3, 10, and 11) waiting time regions. Until the moment of transplantation, the vast majority (93%) of patients with HCC were at home and therefore significantly less often in hospital or ICU than patients without HCC. Accordingly, patients without HCC were more often dependent on life support. Median MELD-Na scores in patients without HCC, with T2 HCC, and with HCC beyond the criteria were 25, 12, and 11, respectively. In addition, 4.2% of the patients had HCC and underwent a transplant based on their MELD(-Na) score, which was higher than their exception score.

**Table 1:** Patient (recipient and donor) characteristics at transplantation between 2010 and 2019.

	No HCC	T2 HCC	HCC outside the criteria	*p*
n	24,503	6,922	5,448	
Age, median (IQR), years	56.0 (48.0–62.0)	60.0 (56.0–65.0)	62.0 (58.0–65.0)	<0.001
Female sex, n (%)	8,926 (36.4)	1,614 (23.3)	1,133 (20.8)	<0.001
Race/ethnicity, n (%)				<0.001
White	18,897 (77.1)	4,907 (70.9)	3,705 (68.0)	
Black	1,956 (8.0)	683 (9.9)	542 (9.9)	
Hispanic	2,790 (11.4)	873 (12.6)	782 (14.4)	
Other	860 (3.5)	459 (6.6)	419 (7.7)	
BMI, median (IQR)	28.0 (25.0–33.0)	28.0 (25.0–32.0)	28.0 (25.0–32.0)	n.s.
Aetiology of disease, n (%)				<0.001
Alcoholic	6,938 (28.3)	—	—	
Cholestatic	2,805 (11.4)	—	—	
HCV	4,666 (19.0)	—	—	
NASH	4,688 (19.1)	—	—	
Other	5,406 (22.1)	—	—	
T2 HCC	—	6,922 (100)	—	
HCC outside the criteria	—	—	5,448 (100)	
Diabetes, n (%)	6,113 (24.9)	2,237 (32.3)	1,863 (34.2)	<0.001
Dialysis dependent, n (%)	20,998 (85.7)	6,803 (98.3)	5,389 (98.9)	<0.001
MELD score, median (IQR)	25.0 (18.0–33.0)	12.0 (9.0–16.0)	11.0 (8.0–14.0)	<0.001
MELD-Na score, median (IQR)	27.0 (20.0–34.0)	13.0 (9.0–17.0)	11.0 (8.0–16.0)	<0.001
Region waiting time,∗ n (%)				<0.001
Long	4,614 (18.8)	1,643 (23.7)	1,401 (25.7)	
Medium	9,135 (37.3)	3,093 (44.7)	2,255 (41.4)	
Short	10,754 (43.9)	2,186 (31.6)	1,792 (32.9)	
Location, n (%)				<0.001
Home	14,142 (57.7)	6,385 (92.2)	5,124 (94.1)	
Hospital	6,423 (26.2)	392 (5.7)	251 (4.6)	
ICU	3,938 (16.1)	145 (2.1)	73 (1.3)	
Life support dependent (%)	2,251 (9.2)	79 (1.1)	39 (0.7)	<0.001
AFP (ng/ml), mean (SD)	—	67 (294)	61 (262)	<0.001
Number of HCC lesions (%)				<0.001
1	—	74.2	65.5	
2	—	19.3	24.6	
3	—	6.5	9.9	
Total tumour diameter (cm), mean (SD)	—	2.79 (1.11)	3.17 (1.89)	<0.001
Donor risk index, median (IQR)	1.35 (1.11–1.64)	1.36 (1.11–1.65)	1.37 (1.11–1.65)	n.s.

AFP, alpha-foetoprotein; HCC, hepatocellular carcinoma; ICU, intensive care unit; MELD-Na, model for end-stage liver disease sodium; MELD, model for end-stage liver disease; NASH, non-alcoholic steatohepatitis; UNOS, United Network for Organ Sharing.

∗ Long wait time is UNOS regions 1, 5, and 9; medium wait time is regions 2, 4, 6, 7, and 8; and short wait time is regions 3, 10, and 11.

The serum alpha-foetoprotein (AFP) concentrations at transplantation for patients with HCC within the Milan/T2 criteria and those with HCC initially outside the Milan/T2 criteria was on average (SD) 67 (294) and 61 (262) ng/ml, respectively. The average AFP levels were higher in patients with T2 HCC than in patients with HCC beyond the criteria, which was as a result of the higher frequency of downstaging non-LT treatment. At the time of transplantation, patients with HCC outside the criteria more frequently had two or three tumours. Average total tumour diameter (SD) for T2 and non-T2 HCC was 2.79 (1.11) and 3.17 (1.89) cm, respectively.

Donor risk index scores were comparable for patients without and with HCC; therefore, patients with HCC on average received the same donor quality organs as patients without HCC.

### Waiting list survival model

The significant predictors of the waiting list Cox model are shown in Table S1. In summary, the most important predictors of survival without LT were age, MELD(-Na) score, serum sodium, serum AFP, serum albumin, presence of diabetes mellitus, presence of ascites, and liver disease aetiology. By correcting coefficients through IPCW, the importance of MELD(-Na) increased (data not shown), which was expected, as we aimed to correct for dependent censoring bias. The waiting list survival prediction model showed excellent discrimination, with a 5-year AUROC of 0.86 (95% CI 0.86–0.86). The CI was small owing to the large size of the cross-sectioned data (22,847,499 rows).

### Post-transplantation survival model

The significant predictors for the post-transplantation survival model are shown in Table S2. The most important were age, liver disease aetiology, being of Black race/ethnicity, presence of diabetes mellitus, mechanical ventilation, total tumour diameter, serum AFP, and donor risk index score. Patients with HCC with MELD(-Na) >19, AFP >24 ng/ml, and total tumour diameter >3.2 cm had the worst post-transplant 5-year survival rates (58.1%; 95% CI 50.2–67.2%). For all other patients with HCC, 5-year survival was above 60% (Fig. S2).^29^ The post-transplant model AUROC of 5-year survival was 61.9 (95% CI 61.2–62.6), indicating respectable discrimination. More importantly,^34^ model calibration was excellent (Fig. S3), which meant that our predicted risks closely resembled observed risks. After establishing model accuracy, survival estimates and benefit were calculated in the validation data.

### Survival without and with LT

**Fig. 2:**
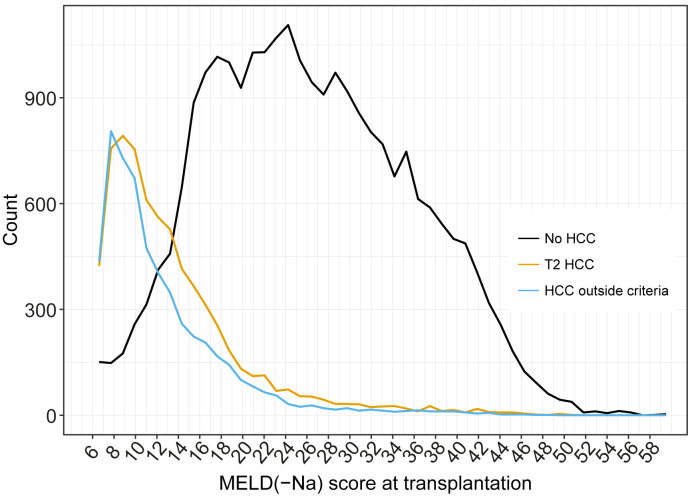
Distribution of MELD(-Na) scores at transplantation, per (non-)HCC disease. Patients without HCC mostly underwent a transplant at MELD(-Na) scores >14. By contrast, patients with HCC mostly underwent a transplant below MELD(-Na) 14. In addition, a significant part of patients without HCC undergo a transplant above MELD(-Na) 30, whereas only 3% of HCC patients undergo a transplant at MELD(-Na) ≥30. HCC, hepatocellular carcinoma; MELD(-Na), model for end-stage liver disease (sodium).

The distribution of MELD(-Na) scores at transplantation is shown in Fig. 2. Patients without HCC mostly underwent a transplant at MELD(-Na) scores above 14, and patients with HCC mostly underwent a transplant below MELD(-Na) 14.

**Fig. 3:**
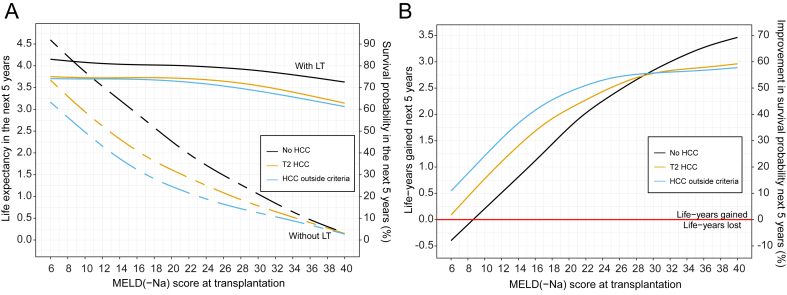
Average 5-year survival and benefit per MELD(-Na) score at transplantation. (A) The mean survival during the next 5 years with and without transplantation per MELD(-Na) score, for the waiting list (dashed lines) and after transplantation (solid lines). Note that the dashed lines represent future ‘without LT’ waiting list survival. The left y-axis shows life expectancy in years, the right y-axis shows survival probability. Thus, for example, a patient without HCC with MELD-Na 22 on the waiting list is expected to live 2 years out of the next 5 years; that is, without transplantation, the survival probability is 40%. Performing a transplant in this patient would result in a life expectancy of 4 years per next 5 years, that is, 80% 5-year survival probability. Please note that these survival probabilities are the mean survival during the next 5 years. This differs from survival probability at 5 years. (B) The survival benefit of liver transplantation during the next 5 years per MELD(-Na) score. The left y-axis shows the average life-years gained in the next 5 years. The right y-axis shows the percentage improvement in mean survival probability during the next 5 years. Thus, for example, a patient with T2 HCC or HCC within the Milan criteria with MELD-Na score 25 will gain 2.5 extra life-years in the next 5 years; that is, the mean survival increases to 50% through transplantation. HCC, hepatocellular carcinoma; LT, liver transplantation; MELD(-Na), model for end-stage liver disease (sodium).

Fig. 3A shows the smoothed average survival probabilities during the next 5 years, both for post-transplantation (with LT, solid lines) and for remaining on the waiting list (without LT, dashed lines). The survival probabilities at 5 years without and with LT are presented in Table S3, which are perhaps more intuitive survival measures for the clinician and patient. However, these hold no information regarding the 5-year survival trajectory.

For patients without HCC below MELD(-Na) 10, that is, a small number of patients (Fig. 2), mean survival probability without LT was better than with LT survival. At equal MELD(-Na) scores, waiting list survival without LT for patients with HCC was notably lower than that for patients without HCC. Survival without LT probabilities converged at high MELD(-Na) scores; that is, mortality could not increase much more. The average survival with LT in both groups declined above approximately MELD(-Na) 24. However, HCC survival decreased more at higher MELD(-Na) scores, most for patients with HCC outside the criteria. This decrease in post-transplant survival was possibly attributable to disease recurrence.

### Survival benefit: life-years gained per 5 years

The 5-year transplantation survival benefit per MELD(-Na) score and per (non-)HCC disease is shown in Fig. 3B and Table 2 [see Table S4 for the averages per MELD(-Na) score]. As an example, for a patient without HCC MELD(-Na) 25, LT would give 2.35-year survival benefit during the next 5 years.

**Table 2:** Liver transplantation 5-year survival benefit per MELD(-Na) score and (non-)HCC disease.

MELD(-Na) score	No HCC			T2 HCC			HCC outside the criteria		
	No. of patients	%	Life-years gained in 5 years per patient	No. of patients	%	Life-years gained in 5 years per patient	No. of patients	%	Life-years gained in 5 years per patient
6–9	539	2.2	−0.12	2,237	32.0	0.39	2,136	39.2	0.39
10–13	1,299	5.3	0.46	2,027	29.6	0.99	1,564	28.7	0.99
14–17	2,867	11.7	1.07	1,273	18.2	1.60	904	16.6	1.60
18–21	3,455	14.1	1.65	601	8.6	2.01	458	8.4	2.01
22–25	3,822	15.6	2.20	322	4.6	2.37	174	3.2	2.37
26–29	3,234	13.2	2.61	175	2.5	2.70	49	0.9	2.70
30–34	3,528	14.4	3.01	126	1.8	2.82	60	1.1	2.82
35–40	5,783	23.6	3.38	189	2.7	2.90	93	1.7	2.90
All patients	24,503	100	2.30	6,992	100	1.19	5,448	100	1.45

HCC, hepatocellular carcinoma; MELD(-Na), model for end-stage liver disease (sodium).

For the 2.2% of patients without HCC who underwent a transplant at MELD(-Na) below 9, benefit was negative, because mean postoperative life expectancy was lower than survival without LT. With increasing MELD(-Na) scores, non-HCC benefit increased approximately linearly, up to 70% mean 5-year survival improvement for MELD(-Na) 40.

The HCC benefit curves flattened with increasing MELD(-Na), whereas non-HCC benefit continued to increase. HCC MELD(-Na) ≥30 benefit estimates should be interpreted carefully, as they represent a small number of patients, that is, 4.5% of the patients with T2 HCC and 2.8% of the patients with HCC outside the criteria. The HCC benefit flattened at higher MELD(-Na) scores because of decreasing post-transplant survival (Fig. 3A). Therefore, the presence of HCC in patients with severe liver failure may be the detrimental and predominant factor influencing survival.

Below MELD(-Na) 30, patients with HCC gained more benefit than patients without HCC at the same MELD(-Na) score, mainly because of the lower expected HCC waiting list survival in the absence of LT. However, patients without HCC were more likely to undergo a transplant at higher MELD(-Na) scores (Fig. 2 and Table 2). Over 50% of patients with HCC underwent a transplant below MELD(-Na) 14, whereas over 50% of patients without HCC underwent a transplant above MELD(-Na) 26. In terms of benefit, most patients with HCC gained 0.10 to 1.96 years from LT, whereas most patients without HCC gained 2.48 to 3.46 years (Table S4). For all patients across all MELD(-Na) scores, patients without HCC gained 2.3 years in the next 5 years through transplantation, patients with T2 HCC gained 1.19 years, and patients with HCC outside the criteria gained 1.45 life-years (Table 2).

Most patients with HCC had low (<25 ng/ml) AFP levels at transplantation. The value of serum AFP did not correlate well with benefit from LT (Figs. S4 and S5). In addition, 0.8% of patients with HCC had AFP >1,000 ng/ml, possibly indicating futile transplants. In patients with progressive disease, median 5-year benefit was 2.3 for patients initially within the Milan criteria and 2.1 for patients initially outside the Milan criteria (Table S5). Bridging of these patients on the waiting list did not change their median benefit, nor did it show a notably different benefit distribution (Fig. S6). This possibly illustrates the inadequacy of the current UNOS data regarding post-transplantation disease recurrence. Total tumour diameter and its change over time also did not seem to correlate well with benefit or with post-transplant survival (Table S2, and Figs S4 and S7).

### Liver transplant benefit scores

Liver transplant benefit scores could be used as a continuous, equalising metric for (non-)HCC LT access. There might be a need to calculate benefit given specific patient characteristics. This is now possible in the online benefit calculator (https://predictionmodels.shinyapps.io/benefit_calculator/). The calculator was based on a secondary regression analysis with only the most important benefit predictors, which showed an optimism corrected R^2^ of 0.93. We therefore assumed that the calculator reliably predicted benefit. Variable importance in regression is summarised in Fig. S4. When predicting benefit, the MELD(-Na) score was by far most important. Next were serum albumin, presence and type of HCC disease, serum sodium levels, and recipient age. Lastly, the online app also allows users to plot mean benefit per MELD-Na and (non-)HCC disease. This can be used to inform clinicians and patients on the expected survival gain from transplantation, for the population included in this study. It also shows for selected patients with HCC which patients without HCC have equal benefit, that is, which patients would compete for transplant based on benefit scores.

## Discussion

Organ allocation aims to equally distribute donor organs to all patients in need. However, inequal LT access exists. As a result, liver allocation has become increasingly relevant and complex. Survival benefit has gained increased attention,^9^^,^^10^^,^^15^^,^^17^^,^^29^ as its optimisation could improve life-years gained from transplantation for all listed patients.^12^ Moreover, considering survival with and without LT based on patient characteristics closely resembles clinical reasoning.

### Findings

The objective of this study was to estimate and compare LT survival benefit for patients with and without HCC in a recent USA waiting list cohort. Our results showed that mean LT survival benefit was positive across all MELD(-Na) scores, except for patients without HCC with MELD(-Na) scores below 9. Patients without HCC gained most life-years from transplantation, as these patients mostly underwent a transplant above MELD(-Na) 26, where benefit was highest. Patients with HCC mostly underwent a transplant below MELD(-Na) 14, which yielded lower survival benefit. Liver function was the most important predictor of benefit. It is now possible online to calculate 5-year survival benefit based on specific patient characteristics (see https://predictionmodels.shinyapps.io/benefit_calculator/).

### Benefit definition

Benefit was defined as the difference in survival with and without LT during the next 5 years. The endpoint of survival analysis was 5 years, because using 10-year or overall survival as outcome would give too much importance to variables that predict post-transplant survival.^4^^,^^29^ In addition, further increasing the prediction horizon made estimates less certain. At 5 years, the waiting list model showed an excellent AUROC, also when compared with other similar analyses.^12^^,^^14^ Compared with recently reported and tested post-transplant survival models, our 5-year post-transplant survival model performed similarly (LiTES) or better (HALT-HCC and Metroticket).^6^

### Estimation of benefit

We choose to estimate benefit from the moment of possible LT. Our methods therefore differed from previous clinical studies that modelled waiting list survival counted from first registration.^9^^,^[15], [16], [17], [18] Our goal was to model future survival without LT, whereas counting from baseline gives survival before LT (Fig. 1). Moreover, patient states at first listing and transplantation should not be compared, as survival changes within each patient over waiting list time owing to, for example, disease progression and possible non-LT treatments.^12^^,^^13^^,^[20], [21], [22], [23] We therefore calculated counterfactual waiting list survival (without LT) through time-dependent analysis with additional correction for bias.^12^^,^^23^ These methods are less often applied than intention-to-treat and competing risk analyses, but this does not mean we should not use them.^35^ Others performed similar analyses over time, but averaged calculated benefit over waiting list follow-up,^12^^,^^14^ which for us seemed suboptimal as possible transplantation and its benefit occurred at one moment in time per patient. Lastly, some previous studies calculated benefit using characteristics of a ‘median donor’ assigned to all patients.^9^^,^^36^ Instead, we choose to use the actual transplantation between 2010 and 2019, with the aim to best evaluate reality, as the observed transplants indicate inequity between patients with and without HCC.^19^ Still, estimated benefit showed resemblance to results in literature, mainly because liver function is the dominant predictor of survival and benefit.

### Non-HCC and HCC benefit

A competing risks study by Berry and Ioannou^9^ showed that patients with HCC in the USA overall gained negative or little benefit from transplantation, that is, that patients with HCC wasted benefit. This contrasts with our findings that mean HCC benefit was positive across all MELD(-Na) scores, mainly because HCC survival without LT was low. Clinically, it makes sense that out of two otherwise identical patients, the patient with HCC will live shorter without LT because of the malignancy *in situ*.^37^ It was suggested that Berry and Ioannou^9^ overestimated HCC waiting list survival^38^ and that having HCC increased risk of waiting list mortality by factor 1.5.^12^ Therefore, on the individual patient level, transplantation for HCC will add life-years. However, on a population level (over)prioritising patients with HCC can indirectly waste benefit, as patients without HCC often will gain more survival from LT owing to worse liver function. Interestingly, many patients with HCC underwent a transplant at MELD(-Na) <10, which was considered harmful in a previous study.^18^ Moreover, resectable HCC may be regarded a contraindication for LT,^4^ especially when considering the limited number of available liver donors. Therefore, the selection of patients with HCC for transplantation remains one of the most important parts of liver graft allocation.^29^

### Using benefit scores

The LT benefit scores offer a continuous metric to stratify survival equally for patients without and with HCC, as one single model is used for both groups. This abandons the use of waiting time, which is inherently flawed,^39^ and binary criteria, which allow underreporting of HCC severity.^40^^,^^41^ Current HCC criteria lack granularity, as patients who have the same waiting list priority can have very different survival with(out) LT.^6^^,^^9^^,^^12^^,^^15^ Changing LT priority based on benefit scores could therefore prevent loss of life-years, as also shown in simulations.^12^ Allocation policies such as the HCC cap, HCC delay, and Median MELD at Transplant helped reduce HCC LT access, but patients with HCC are currently still better of regarding waiting time, transplantation rates, and death rates.^8^^,19^ Clearly, there is a need for an equalising principle for all eligible LT candidates. Still, consensus must be reached whether to consider benefit in allocation at all. Understandably, some feel uncomfortable to base treatment decisions on future post-transplant outcomes, which is in part why USA policy first focused on improving regional disparities.^26^^,^^42^^,^^43^ By contrast, there is consensus on acceptable post-transplant outcomes,^44^ and post-transplant survival can be accurately predicted. Interestingly, in the UK, a benefit-based allocation system was implemented in 2018.^45^ The evaluation of this system will be valuable for the debate on benefit and its role in liver allocation. However, it is most important that, regardless of the driving allocation principle, scarce liver grafts should be fairly distributed based on patient characteristics and disease severity, not on arbitrary exception points.

### Limitations

Our study has limitations. We excluded patients, for example, those with exception points who did not have HCC, and therefore, for these patients, our findings should not be applied to estimate transplant benefit. However, our goal was to compare patients without and with HCC. In addition, 5-year post-transplant follow-up was not complete for all patients, as we compromised completeness and study period. Within the studied period, allocation policy changed. In the models, only the most relevant changes according to the OPTN were considered. Therefore, smaller policy changes could have influenced our findings by an uncertain degree. Furthermore, we could only draw conclusions based on patients that were listed for transplantation. Therefore, selection bias exists, which is inherent to the analysis of registries. The UNOS also does not register HCC recurrence, which would be valuable as HCC recurrence rates can be up to 20%, after which median survival is less than 1 year.^41^ Our data showed that high-risk patients still underwent a transplant, which could be because of individual patient characteristics beyond the Scientific Registry of Transplant Recipients data, patient wishes, waiting list dynamics at the time of liver graft offering, and experience of the transplantation professionals involved. Studying recurrence data in patients with HCC with MELD >30 would be especially interesting. Still, overall mortality is considered free from bias, whereas disease-specific survival is not.^46^ In addition, owing to the small number of transplants in patients with HCC with MELD(-Na) >30, estimates were less reliable for that group. Lastly, patient survival was used as the main metric. In our view, quality of life (life within the years) should be prioritised when guiding patients and relatives through the clinical decision-making surrounding liver transplantation.

### Conclusions

In conclusion, on an individual level, performing a transplant in patients with HCC resulted in survival benefit. However, on a population level, benefit was indirectly wasted, as patients without HCC were likely to gain more survival owing to decreased liver function. Liver transplant benefit scores offer equal survival stratification for patient with and without HCC. It is now possible online to calculate these scores based on individual patient characteristics. Considering benefit better resembles clinical reasoning and can optimise life-years gained for the whole waiting list population. Survival benefit scores could therefore serve to more equally allocate scarce liver grafts among patients eligible for transplantation.

## Financial support

The manuscript was not prepared by or funded in any part by a commercial organisation. No financial support or grants were used for the preparation of this manuscript.

## Authors’ contributions

Contributed to the design of the study: BG, HP, BH, AB. Contributed to data acquisition: BG. Contributed to data analysis: BG, IP, HP. Were involved in interpretation of the data, and drafting and revising the manuscript: all authors. Approved the final version of the manuscript for submission: all authors.

## Data availability statement

The data are publicly available from OPTN/UNOS, but the authors are not able to share the data owing to restrictions in the data use agreement.

## Conflicts of interest

The authors of this manuscript have no conflict of interest to disclose.

Please refer to the accompanying ICMJE disclosure forms for further details.
