# Supplementary material for: Survival benefit from liver transplantation for patients with and without hepatocellular carcinoma
Source: JHEP Rep. 2023 Sep 13;5(12):100907. doi: 10.1016/j.jhepr.2023.100907 (PMC10685016; doi:10.1016/j.jhepr.2023.100907)

Survival benefit from liver transplantation for patients with and without hepatocellular carcinoma

Ben F.J. Goudsmit, Ilaria Prosepe, Maarten E. Tushuizen, Vincenzo Mazzaferro, Ian P.J. Alwayn, Bart van Hoek, Andries E. Braat, Hein Putter

Table of contents

Supplement 1 2

Table S1 4

Table S2 6

Table S3 7

Table S4 8

Table S5. 9

Supplementary figure legends 10

Fig. S1 11

Fig. S2 11

Fig. S3 13

Fig. S4 14

Fig. S5. 15

Fig. S6. 16

Fig. S7. 17

Supplement 1: in-depth statistical methods to calculate waiting list survival and model performance

The waiting list survival was estimated following the methodology described by Gong and Schaubel[1] (2013). They proposed the use of a partly conditional method to create a model that correctly mimics the need to estimate survival at specific moments in calendar time on cross-sections of patients rather than on follow-up time points on a cohort of patients. In their work, a set on evenly spaced calendar dates (cross-sections) is created and survival is estimated from those times onwards. Moreover they suggested some valid adaptations of IPCW to correct for the dependent censoring of transplanted patients in the created framework.

In short, based on observed waiting list data, we calculated patient survival in absence of treatment, much like the control group of a randomized controlled trial (RCT).

In order to avoid excessive computational burden, we opted for biweekly cross-sections. Patients were randomly assigned to train data and test data, with a percentage of 67% and 33% respectively. In particular, in order to preserve these percentages in the post-transplantation model, we first randomly split the transplanted patients into 67% train and 33% test and then we widened the two groups with, respectively, the 67% and 33% of patients who never received transplantation.

At each cross-section *k* (CS_k), for each patient *i* having covariates $Z_i(t)$, weights for IPCW were estimated following the indication of the paper for the type B weights:

$$W_{ik}^{B}(t) = Y_{ik}\epsilon_{ik}\frac{\exp\{-\Lambda_{ik}^{T}(t)\}}{\exp\{-\Lambda_{i}^{T}(S_{ik}+t)+\Lambda_{i}^{T}(S_{ik})\}}$$

where Y_{ik} is an indicator that takes value 1 if patient *i* has not been transplanted yet at cross-section *k* and 0 otherwise, ε_{ik} is an indicator that takes value 1 if patient *i* is active at cross-section *k* and 0 otherwise, S_{ik} is the time from *i*-patient's first eligibility to cross-section *k*, and $\Lambda_i^T(t)$ and $\Lambda_{ik}^T(t)$ are the cumulative hazard of the treatment models $\Lambda_i^T(t) = \int_0^t \epsilon_i(u)\lambda^T(u)\exp\{\theta_0 Z_i(u)\}du$ and $\Lambda_{ik}^T(t) = \int_0^t \epsilon_{ik}(u)\lambda_k^T(u)\exp\{\theta_1 Z_i(S_{ik})\}du$.

These two partly conditional hazard regression models, used to track transplantation chances at each time-point, were estimated using the whole population. In fact, given the choice to divide patients into train and test data randomly, we have assumed that the underlying transplantation model would be the same in the two groups.

The waiting list model was estimated as a weighted partly conditional hazard regression as recommended in the Gong and Schaubel paper, with hazard

$$\lambda_{0k}(t; s|Z_i(S_{ik}), \epsilon_{ik} = 1) = \lambda_k(t)\exp\{\beta Z_i(S_{ik})\}.$$

Survival benefit was then defined as the life-years gained from the moment of transplantation during the next five years (Figure 1).[12,29] Survival benefit was calculated as the difference between the observed posttransplant survival and patient survival on the waiting list survival (described here above) had the patient not been transplanted.

Briefly, we did not use intention-to-treat (ITT) or competing risk analysis, because 1) we wanted to best approximate a RCT setting, 2) wanted to prevent underestimation of mortality and subsequent

undertreatment,[33] and 3) the intention was to model changes in waiting list disease over time beyond the moment of first listing.

Table S1: waiting list survival model summary

Predictor	coefficient	HR	low95	up95	p
Age	0.035	1.04	1.03	1.04	<0.001
Female sex	0.100	1.10	1.03	1.18	0.005
ABO – O	ref	ref	ref	ref	
ABO – A	0.017	0.983	0.913	1.058	0.647
ABO – AB	0.28	1.322	1.027	1.704	0.031
ABO – B	0.124	0.884	0.782	0.999	0.048
Race White	ref	ref	ref	ref	
Race Black	-0.17	0.843	0.741	0.96	0.01
Race Hispanic	0.024	0.976	0.887	1.073	0.618
Race Other	0.154	0.857	0.705	1.041	0.12
Disease Other	ref	ref	ref	ref	
Disease ALD	-0.19	0.827	0.754	0.907	<0.001
Disease HCV	0.179	0.836	0.759	0.922	<0.001
Disease HBV	0.594	0.552	0.376	0.811	0.002
Disease HCC	0.298	0.742	0.624	0.883	0.001
Diabetes	0.145	1.16	1.07	1.24	<0.001
Albumin	-0.522	0.59	0.56	0.63	<0.001
Ascites None	ref	ref	ref	ref	
Ascites Slight	0.093	1.10	1.00	1.20	0.039
Ascites Moderate	0.210	1.23	1.10	1.39	<0.001
MELD(-Na)	0.070	1.07	1.06	1.09	<0.001
log(42-MELD(-Na))	-0.820	0.44	0.36	0.54	<0.001
Sodium	-0.049	0.95	0.94	0.96	<0.001
CPS grade A	ref	ref	ref	ref	
CPS grade B	0.054	1.055	0.954	1.167	0.295
CPS grade C	0.186	1.205	1.06	1.369	0.004
Log (AFP + 1)	0.194	1.21	1.17	1.27	<0.001
Log (TTD +1)	0.134	1.14	1.02	1.28	0.024
AFP difference with previous	0.155	1.17	1.06	1.29	0.002
Exception for HCC outside policy	0.452	1.57	1.33	1.86	<0.001
Exception * MELD(-Na) interaction	-0.054	0.95	0.91	0.98	0.005
Policy exceptions 2005-03-16	ref	ref	ref	ref	
Policy exceptions 2015-10-08	0.254	1.29	1.03	1.61	0.026
Policy exceptions 2017-12-12	0.389	1.47	1.11	1.97	0.008
Policy 2015-10-08 * waiting time interaction	0.063	1.07	1.00	1.13	0.043
Policy 2017-12-12* waiting time interaction	0.073	1.08	1.00	1.15	0.043
Time of cross-section spline df 1	0.030	1.03	0.89	1.19	0.675
Time of cross-section spline df 2	-0.146	0.86	0.73	1.02	0.079
Time of cross-section spline df 3	-0.076	0.93	0.81	1.07	0.288
Time of cross-section spline df 4	-0.178	0.84	0.62	1.12	0.234

Time of cross-section spline df 5	-0.312	0.73	0.62	0.87	<0.001

AFP: alpha-fetoprotein in ng/mL, TTD: total tumor diameter in cm, MELD(-Na): model for end-stage liver disease (sodium) score, HCC: hepatocellular carcinoma

Table S2: post-transplant survival model summary

Predictor	coefficient	HR	low95	up95	p
Recipient age spline df1	0.131	1.14	0.82	1.59	0.437
Recipient age spline df2	0.556	1.74	1.36	2.24	<0.001
Recipient age spline df3	0.567	1.76	0.81	3.83	0.152
Recipient age spline df4	1.060	2.89	1.72	4.86	<0.001
Disease ALD	ref	ref	ref	ref	ref
Disease HCV	0.238	1.27	1.13	1.42	<0.001
Disease NASH	-0.062	0.94	0.83	1.07	0.348
Disease Other	-0.055	0.95	0.85	1.06	0.332
Disease T2 HCC	-0.329	0.72	0.61	0.85	<0.001
Disease not T2 HCC	-0.244	0.78	0.66	0.93	0.006
Race Other	ref	ref	ref	ref	ref
Race White	0.134	1.14	0.98	1.34	0.099
Race Black	0.395	1.48	1.24	1.78	<0.001
Race Hispanic	-0.021	0.98	0.82	1.18	0.825
Diabetes	0.248	1.28	1.19	1.38	<0.001
Dialysis	0.215	1.23	1.10	1.40	<0.001
Ventilated	0.522	1.69	1.43	1.99	<0.001
Location home	ref	ref	ref	ref	ref
Location hospital	0.191	1.21	1.09	1.34	<0.001
Location ICU	0.251	1.29	1.11	1.49	<0.001
Total tumor diameter	0.062	1.06	1.04	1.08	<0.001
log(AFP + 1)	0.174	1.19	1.15	1.23	<0.001
DRI	0.285	1.33	1.22	1.44	<0.001

ALD: alcoholic liver disease, HCV: hepatitis C virus induced cirrhosis, NASH: non-alcoholic steatohepatitis, HCC: hepatocellular carcinoma, ICU: intensive care unit, AFP: alpha-fetoprotein, TTD: total tumor diameter in cm, MELD(-Na): model for end-stage liver disease (sodium) score

Table S3: Survival probability without and with LT *at* five years

Survival probability at five years (%)							
Without LT				With LT			
MELD(-Na)	non-HCC	T2 HCC	HCC outside criteria	MELD(-Na)	non-HCC	T2 HCC	HCC outside criteria
6	83.6	52.8	39.8	6	89.1	82.1	82.1
7	80.9	48.7	33.9	7	88.7	81.9	81.0
8	74.8	42.3	29.6	8	87.2	81.6	80.7
9	60.5	35.9	25.6	9	86.7	81.6	80.7
10	57.9	33.0	21.7	10	86.4	81.7	80.9
11	54.0	26.7	20.8	11	86.3	80.6	81.3
12	47.8	25.2	15.5	12	85.9	80.6	81.7
13	45.9	23.0	13.2	13	86.8	82.2	80.3
14	39.7	18.8	9.7	14	85.9	82.0	81.6
15	36.5	14.6	10.4	15	86.4	81.2	81.3
16	31.0	16.5	10.1	16	85.8	81.4	81.3
17	28.8	11.3	5.3	17	85.9	82.1	80.3
18	24.7	11.0	5.2	18	85.8	82.1	80.2
19	21.7	7.6	5.9	19	86.2	80.1	81.3
20	17.7	7.8	7.9	20	85.9	81.5	81.5
21	16.1	11.0	3.2	21	85.8	81.0	79.6
22	14.1	8.6	2.9	22	86.0	81.8	80.1
23	11.1	2.8	0.8	23	85.5	80.8	78.4
24	10.4	2.9	2.7	24	85.1	82.0	81.3
25	8.5	4.0	0.9	25	84.8	78.6	82.4
26	6.8	3.8	0.7	26	84.9	79.7	73.6
27	5.9	2.1	0.1	27	85.2	81.6	79.9
28	4.9	1.0	5.9	28	84.0	79.9	82.9
29	3.6	0.1	0.1	29	84.8	81.1	81.0
30	2.5	2.9	0.7	30	84.6	80.6	69.6
31	1.8	1.2	1.0	31	82.8	78.2	83.3
32	1.9	0.2	0.4	32	83.0	77.3	77.7
33	1.4	0.6	0.0	33	83.1	78.1	71.1
34	0.4	0.0	0.0	34	82.2	72.4	77.8
35	0.7	0.1	2.5	35	82.8	80.2	83.4
36	0.2	0.0	0.0	36	81.3	78.7	70.6
37	0.1	0.0	0.0	37	81.4	68.9	72.1
38	0.0	0.0	0.0	38	80.5	69.3	70.2
39	0.0	0.0	0.0	39	80.4	72.2	72.9
40	0.0	0.0	0.0	40	80.0	72.8	70.0

Table S4: Mean benefit per MELD(-Na) score per (non-)HCC

	No HCC		T2 HCC		outside T2 HCC	
MELD(-Na)	mean benefit	number	mean benefit	number	mean benefit	number
6	-0.32	114	0.12	333	0.57	315
7	-0.27	120	0.24	600	0.70	612
8	-0.20	126	0.42	633	0.88	666
9	0.19	165	0.65	621	1.06	558
10	0.25	225	0.79	546	1.23	552
11	0.35	318	0.97	582	1.33	405
12	0.51	354	1.02	453	1.58	306
13	0.64	378	1.21	444	1.63	312
14	0.83	441	1.45	384	1.96	261
15	0.96	735	1.63	333	1.93	255
16	1.14	831	1.55	321	1.96	177
17	1.23	822	1.94	210	2.24	219
18	1.42	858	1.96	210	2.32	135
19	1.57	876	2.02	156	2.31	126
20	1.75	822	2.13	129	2.23	96
21	1.87	846	1.95	96	2.55	102
22	2.02	936	2.18	135	2.39	51
23	2.19	933	2.50	69	2.69	57
24	2.23	999	2.60	60	2.67	36
25	2.36	912	2.44	54	2.94	33
26	2.48	819	2.36	63	2.44	21
27	2.57	789	2.85	36	2.98	9
28	2.59	756	2.80	39	2.75	9
29	2.80	828	3.09	30	2.98	12
30	2.91	792	2.75	33	2.56	27
31	2.94	636	2.90	27	3.02	9
32	2.98	792	2.89	33	2.90	12
33	3.07	714	2.68	21	2.62	9
34	3.19	543	2.87	9	3.05	6
35	3.25	753	3.09	12	2.74	6
36	3.27	624	3.05	15	2.65	15
37	3.36	549	2.64	30	2.87	9
38	3.37	591	2.80	18	2.85	9
39	3.41	486	2.89	21	2.99	9
40	3.45	2697	2.96	87	2.86	45

Table S5: Median benefit per total tumor burden progression and bridging.

Median 5-year benefit from transplantation

	baseline		ttb 0		ttb stable		ttb increase		ttb decrease	
HCC	T2	outside	T2	outside	T2	outside	T2	outside	T2	outside
downstaged	1,6	1,0	1,9	2,3	1,9	1,8	2,3	2,1	1,6	2,2
only LT	1,9	1,6	1,9	3,0	2,3	2,1	2,3	2,1	1,6	2,2

Number of patients stratified above

	baseline		ttb 0		ttb stable		ttb increase		ttb decrease	
HCC	T2	outside	T2	outside	T2	outside	T2	outside	T2	outside
downstaged	232	161	9	316	302	161	209	209	797	382
only LT	185	54	12	149	271	56	62	56	197	121

Supplementary figure legends

1. The in- and exclusion flowchart.
2. Post transplantation survival plots of HCC patients, stratified based on three variables: MELD-Na score, serum AFP, and tumor diameter. The variable cut-offs are based on the most significant effect in the data. A high MELD-Na score is above 19, a high AFP level is above 24 ng/mL, and a high total tumor diameter is above 3.7 cm. Five-year postoperative survival is worst (<60%) for HCC patients with MELD-Na>19, AFP>24, and diameter >3.7.
3. Calibration plot of post-LT survival model. The blue line shows the estimated calibration, corrected for overfitting. The post-transplant predicted risks match the observed risks very well. Therefore, estimates are reliable.
4. Two layers from the decision tree algorithm used to predict benefit in secondary analysis. For each layer, the decision tree chooses the most relevant cut-off point in the data, e.g., the most important distinction is based on liver disease, and then MELD(-Na) score. Within each 'node' the number and average benefit is shown. The final decision tree used in this study has eight layers.
5. The relation between serum AFP levels at transplantation and 5-year benefit scores in HCC patients. Of note, because of the shape of the distribution, AFP levels are capped at 100 ng/mL, showing 95% of patients.
6. The distribution of benefit scores in HCC patients with varying changes in total tumor burden [TTB] (sum of HCC diameters) and pre-LT on the waiting list. The difference between TTB at transplantation and listing is used. Baseline refers to HCC patients with only one available TTB measurement. TTB 0 are patients coded in the SRTR data with diameter 0 cm. TTB stable, increase and decrease refer to TTB changes since listing.
7. The relation between changes in TTB from listing to transplantation and 5-year benefit scores. Of note: negative values correspond to a smaller TTB at transplantation as compared to listing.

Fig. S1: In- and exclusion chart

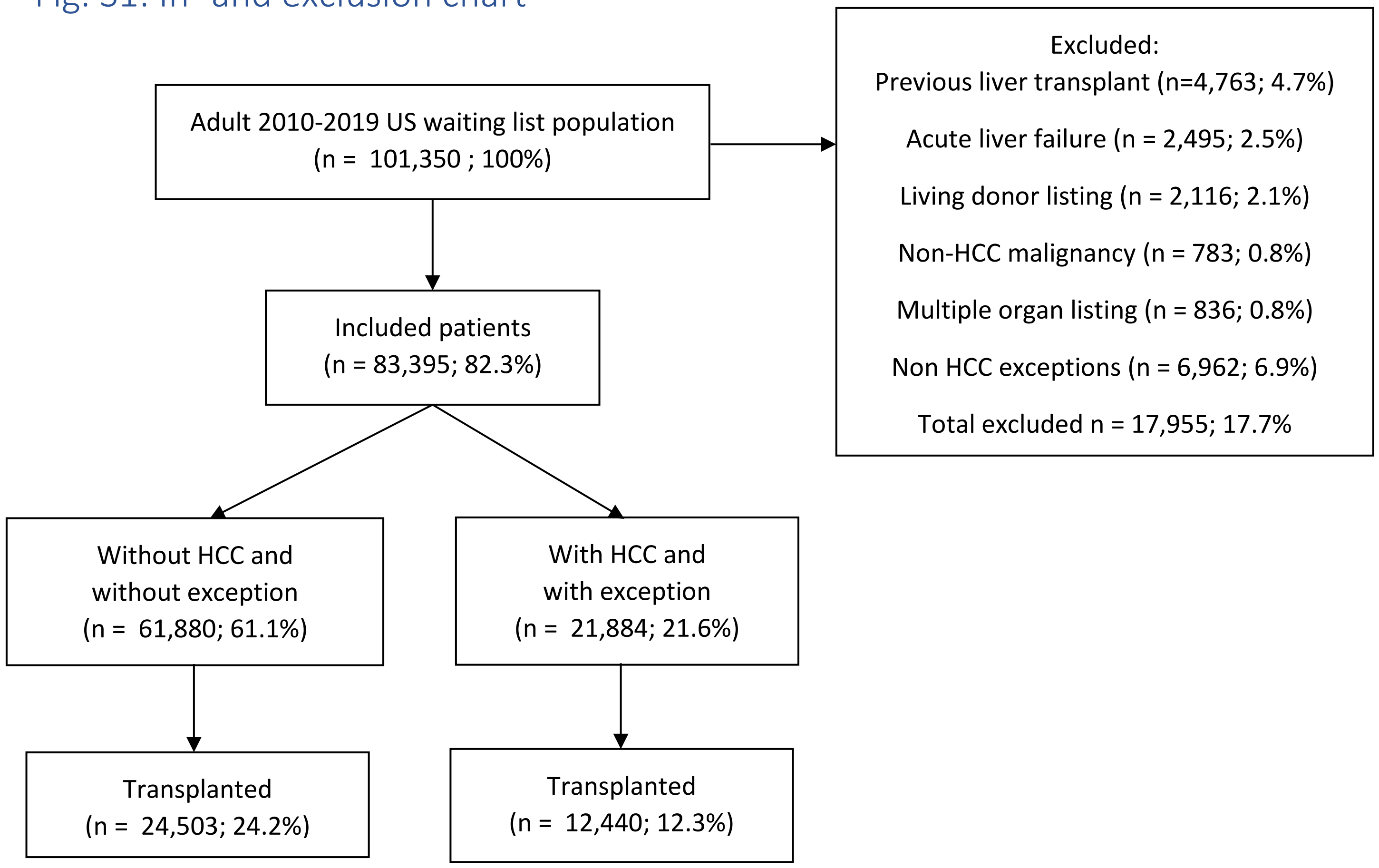

Fig. S2: post transplantation survival stratified for MELD(-Na) score, AFP and total tumor diameter

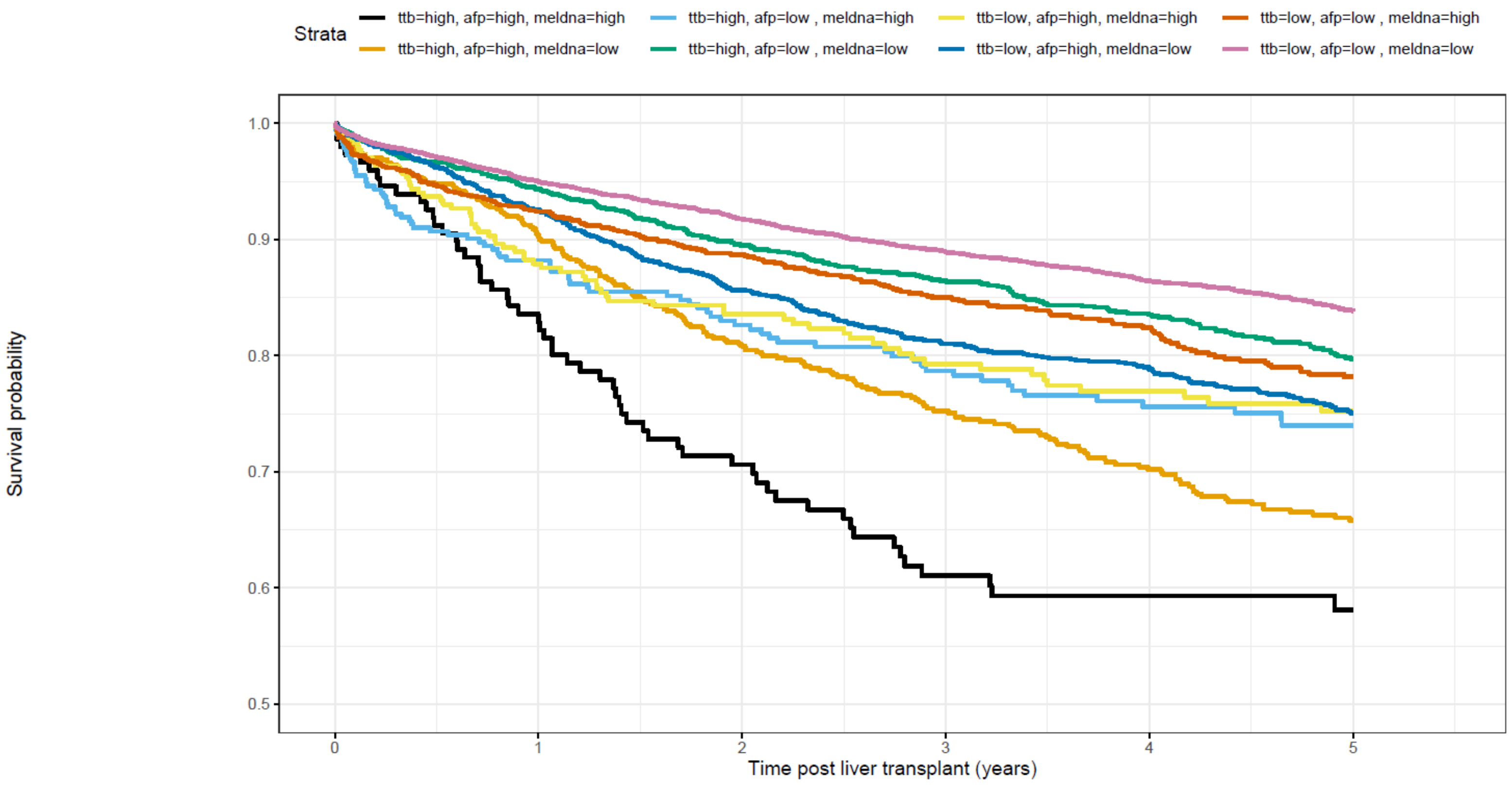

Number at risk

Strata	0	1	2	3	4	5
ttb=high, afp=high, meldna=high	148	118	93	72	62	46
ttb=high, afp=high, meldna=low	671	582	475	399	341	271
ttb=high, afp=low , meldna=high	334	267	225	188	156	124
ttb=high, afp=low , meldna=low	1458	1300	1107	954	762	571
ttb=low, afp=high, meldna=high	303	251	212	175	149	113
ttb=low, afp=high, meldna=low	1637	1440	1198	993	819	619
ttb=low, afp=low , meldna=high	1180	1002	830	651	508	365
ttb=low, afp=low , meldna=low	6639	5685	4526	3560	2702	1899

Time post liver transplant (years)

Fig. S3: post-transplantation model calibration

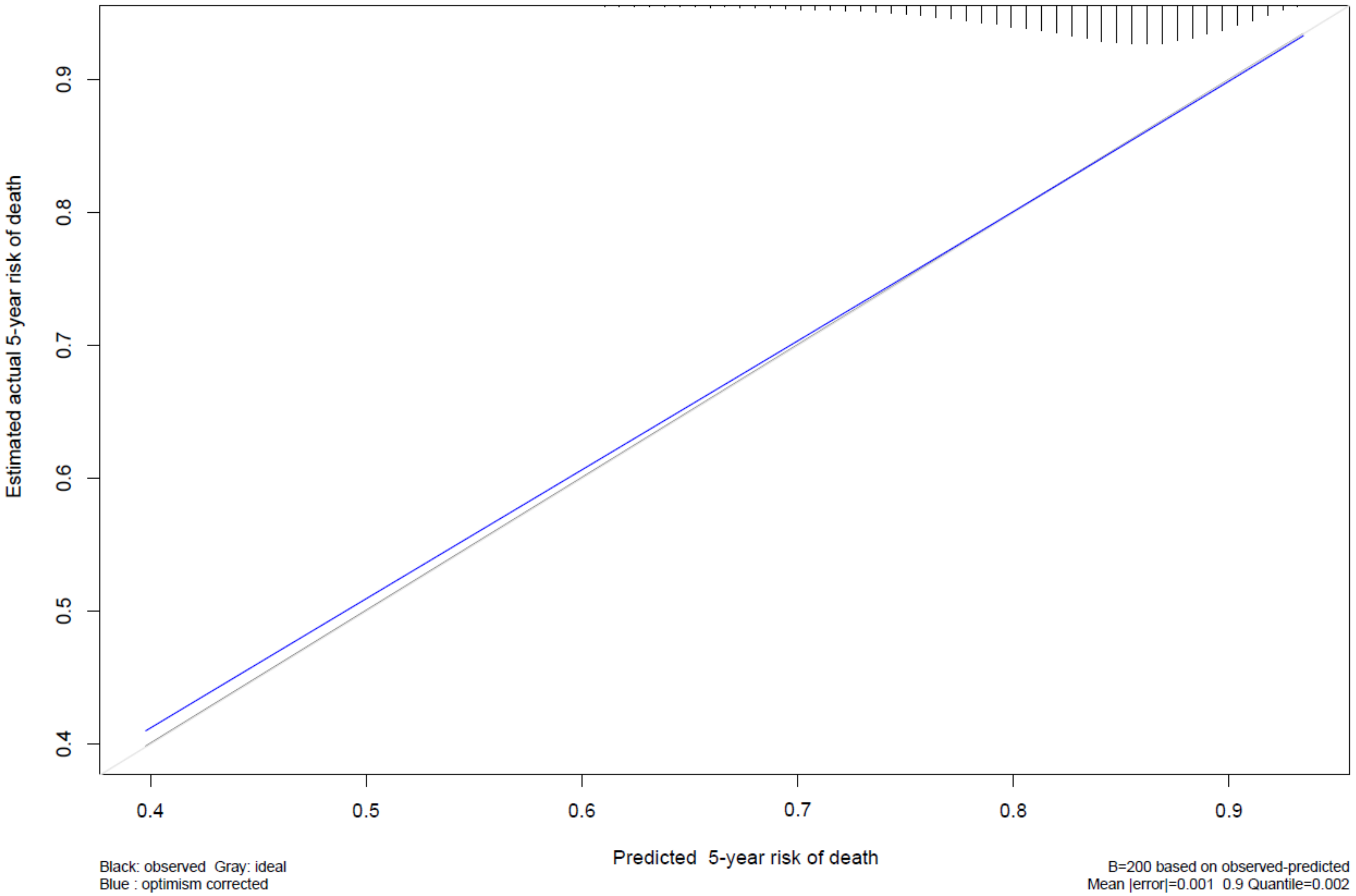

Fig. S4: Variable importance in benefit regression

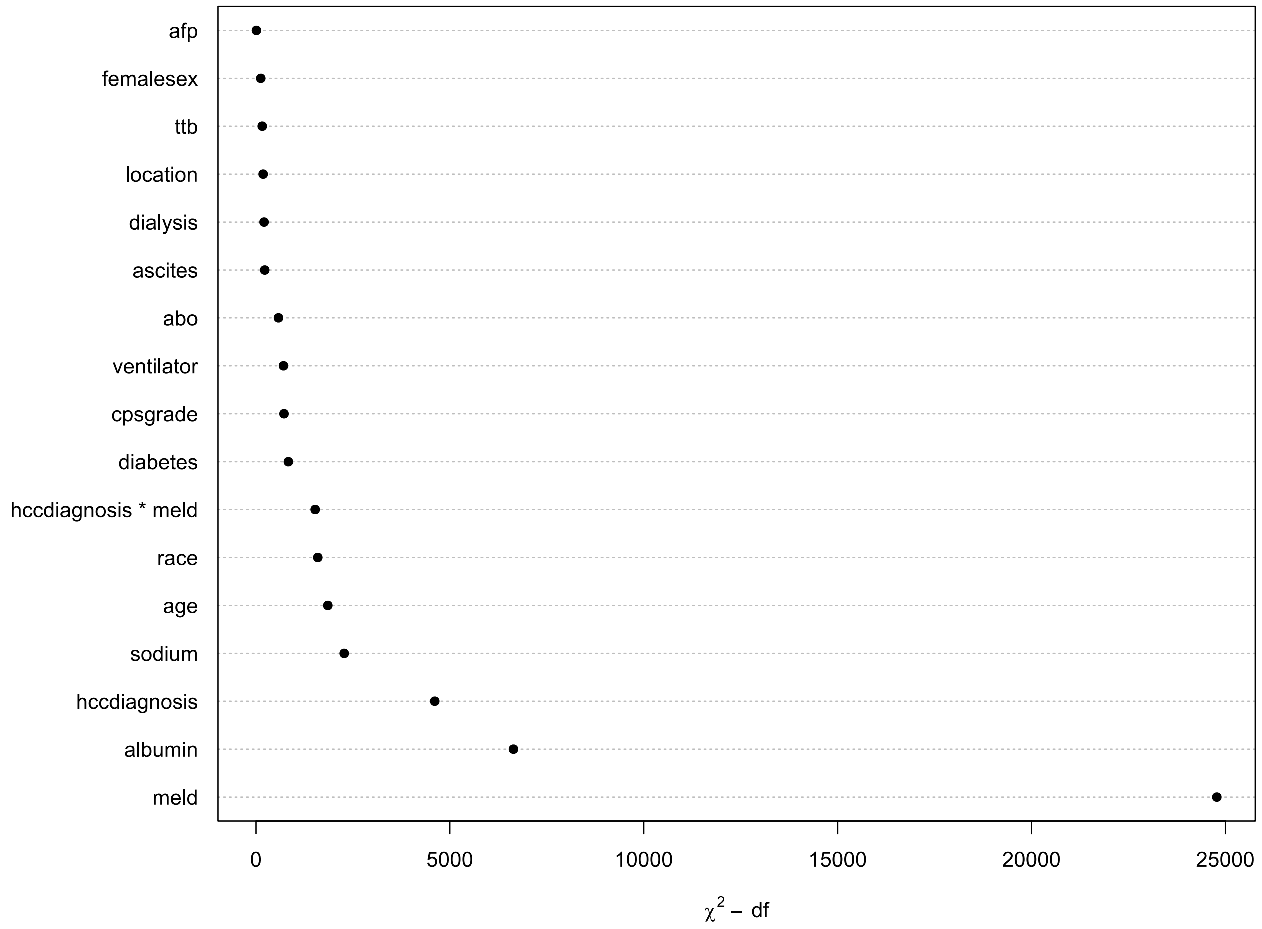

Fig. S5: 5-year benefit and AFP at transplantation.

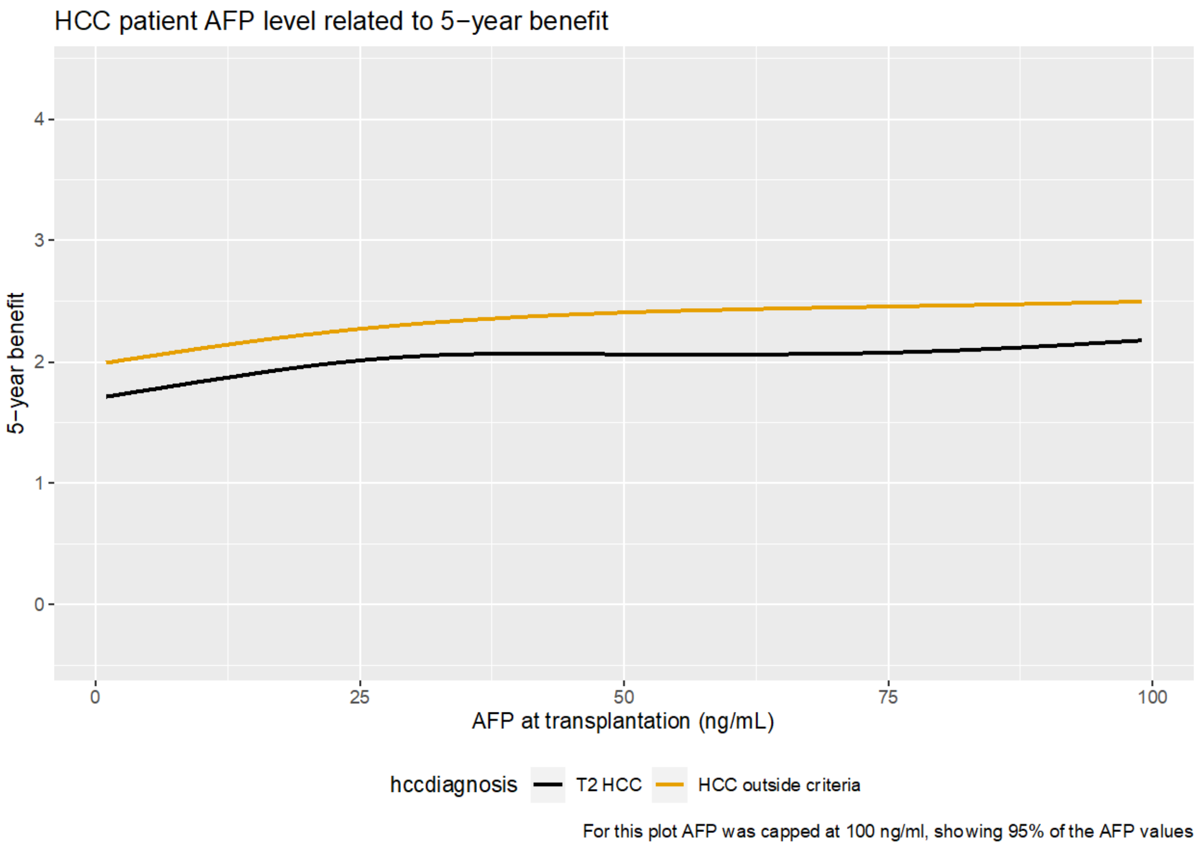

Fig. S6: Benefit distribution per total tumor burden progression and bridging.

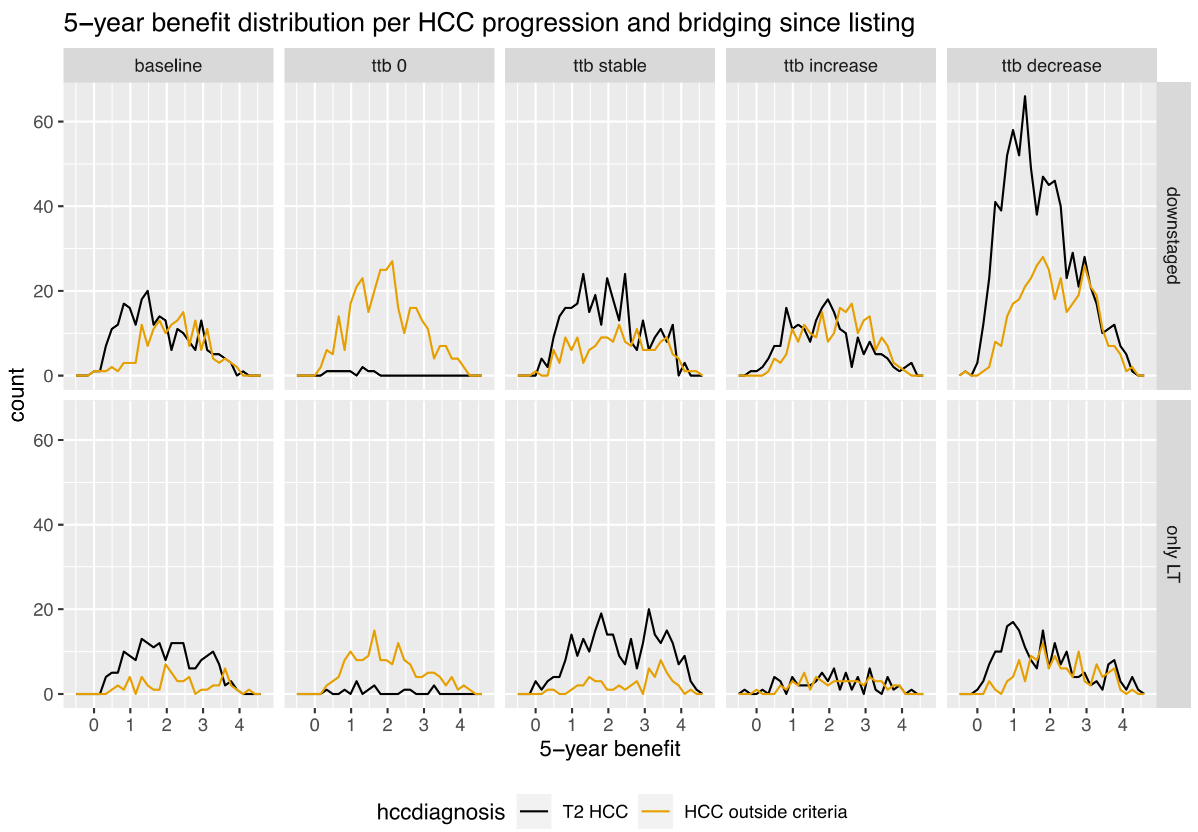

Fig. S7: Benefit per total tumor burden change from listing to transplantation.

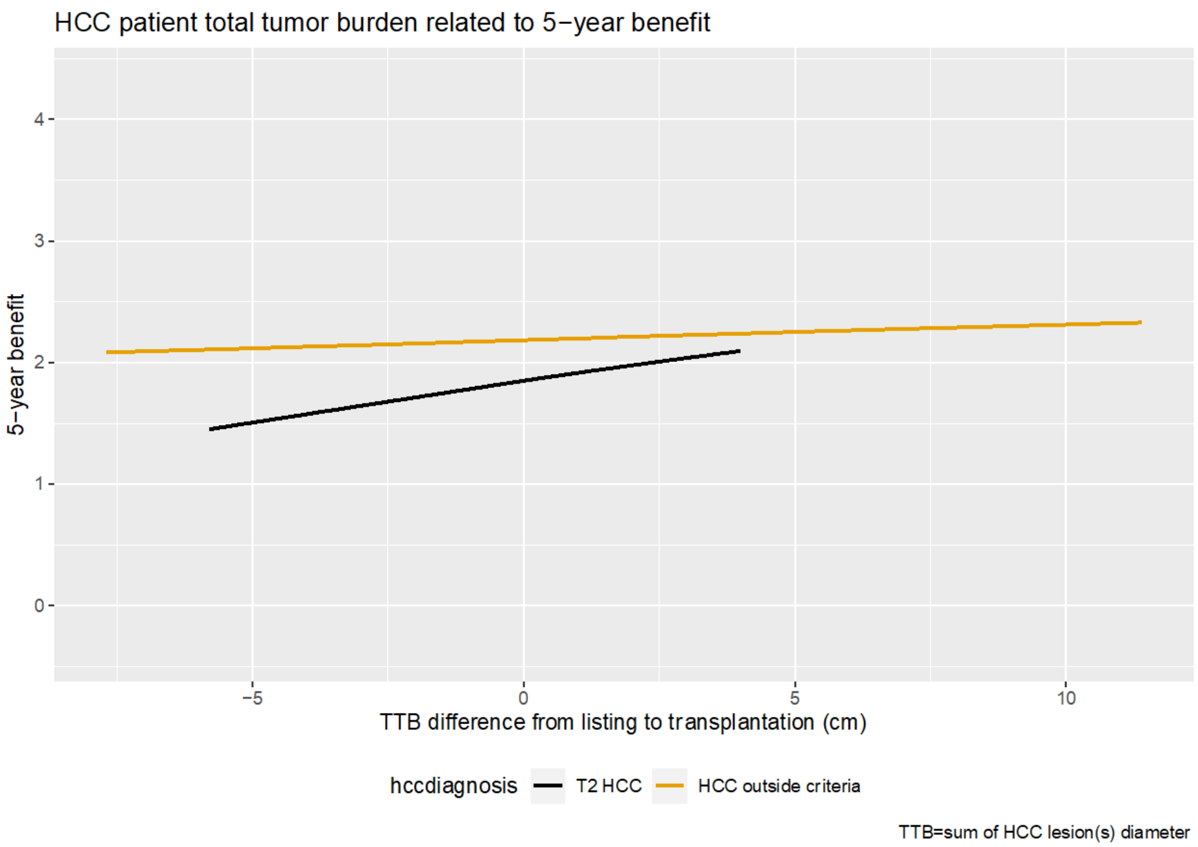

Supplement: Multimedia component 1 [file mmc1.pdf]
